# Shared polygenic risk for ADHD, executive dysfunction and other psychiatric disorders

**DOI:** 10.1038/s41398-020-00872-9

**Published:** 2020-06-09

**Authors:** Suhua Chang, Li Yang, Yufeng Wang, Stephen V. Faraone

**Affiliations:** 1 Peking University Sixth Hospital, Peking University Institute of Mental Health, NHC Key Laboratory of Mental Health (Peking University), National Clinical Research Center for Mental Disorders (Peking University Sixth Hospital), Beijing, China; 2 Research Unit of Diagnosis and Treatment of Mood Cognitive Disorder (2018RU006), Chinese Academy of Medical Sciences, Beijing, China; 3 Departments of Psychiatry and of Neuroscience and Physiology, SUNY Upstate Medical University, Syracuse, NY 13210 USA

**Keywords:** Clinical genetics, ADHD

## Abstract

Many psychiatric disorders are associated with impaired executive functioning (EF). The associated EF component varies by psychiatric disorders, and this variation might be due to genetic liability. We explored the genetic association between five psychiatric disorders and EF in clinically-recruited attention deficit hyperactivity disorder (ADHD) children using polygenic risk score (PRS) methodology. Genome-wide association study (GWAS) summary data for ADHD, major depressive disorder (MDD), schizophrenia (SZ), bipolar disorder (BIP) and autism were used to calculate the PRSs. EF was evaluated by the Stroop test for inhibitory control, the trail-making test for cognitive flexibility, and the digital span test for working memory in a Chinese ADHD cohort (*n* = 1147). Exploratory factor analysis of the three measures identified one principal component for EF (EF-PC). Linear regression models were used to analyze the association between each PRS and the EF measures. The role of EF measures in mediating the effects of the PRSs on ADHD symptoms was also analyzed. The result showed the PRSs for MDD, ADHD and BIP were all significantly associated with the EF-PC. For each EF component, the association results were different for the PRSs of the five psychiatric disorders: the PRSs for ADHD and MDD were associated with inhibitory control (adjusted *P* = 0.0183 and 0.0313, respectively), the PRS for BIP was associated with working memory (adjusted *P* = 0.0416), and the PRS for SZ was associated with cognitive flexibility (adjusted *P* = 0.0335). All three EF measures were significantly correlated with ADHD symptoms. In mediation analyses, the ADHD and MDD PRSs, which were associated with inhibitory control, had significant indirect effects on ADHD symptoms through the mediation of inhibitory control. These findings indicate that the polygenic risks for several psychiatric disorders influence specific executive dysfunction in children with ADHD. The results helped to clarify the relationship between risk genes of each mental disorder and the intermediate cognitive domain, which may further help elucidate the risk genes and motivate efforts to develop EF measures as a diagnostic marker and future treatment target.

## Introduction

Executive functioning (EF) is a high-order set of cognitive functions that regulate an individual’s capacity to change and adjust his or her behaviors according to the shifting demands of complex environments^1^. EF dysfunction is associated with many psychiatric disorders, such as attention deficit hyperactivity disorder (ADHD), major depressive disorder (MDD), bipolar disorder (BIP), autism (ASD), and schizophrenia (SZ)^2–5^. EF encompasses multiple processes^6,7^. Although most psychiatric disorders are associated with EF dysfunction, different neuropsychiatric disorders showed disorder-specific EF deficits^8,9^; for example, the largest effect sizes were observed for response inhibition in individuals with ADHD^10^, for verbal and nonverbal memory in individuals with BIP^11^, and for cognitive flexibility in individuals with SZ^12^. These results suggest that specific EF components might be good markers for specific types of psychopathology. Understanding the role of separate EF components in psychiatric disorders is a critical step in gaining a better understanding of disease psychopathology. Currently, the specificity of these EF component deficits in different psychiatric disorders is not clear^13^.

EF is heritable. Similar EF performance has been observed in family members and twins. The heritability of EF was calculated to be approximately 20 to 40%^14^. The association between EF dysfunction and the psychiatric diseases may, in part, reflect pleiotropy, which is the overlap between the genetic liability of two or more traits, perhaps owing to shared causal pathways^15^. Thus, the genes that influence vulnerability to psychiatric disorders may also influence EF deficits.

It has been widely acknowledged that many psychiatric disorders share genetic etiology^16–18^. Most of the studies investigated shared genetics among a variety of disorders. Only a few studies have discussed the relationship between genetic risk of disorders and intermediate phenotypes. Shared genetic risks have been reported between SZ^19,20^, depression^21^, ADHD^22^ and decreased general cognitive ability^17^, autism and increased cognitive ability^23^, while BIP had mixed results^17,24^. Polygenic risk score (PRS) of ADHD has been associated with components of EF: working memory, vigilance arousal, but not inhibitory control, verbal-numerical reasoning^25–27^. The research for the genetic association of other disorders with specific component was limited or inconsistent^28,29^. The Brainstorm project identified several correlations between psychiatric disorders and intelligence, cognition and education^17^, but not specific EF components. Benca et al. examined the PRSs of five psychiatric disorders (i.e., autism, ADHD, BIP, MDD, and SZ) with three EF components (mainly involving the shifting component) in a small sample of twins, but failed to find any significant results^30^.

The reported studies showed inconsistent results for the genetic association between polygenic risk score of psychiatric disorders with cognition. Most of the cognition data of previous studies used population-based samples, whose phenotype distribution may differ with clinical-based samples. Sample difference in ages also yielded inconsistent results. For example, polygenic risk score of ADHD was associated with cognition in children samples^31^ but not in adult sample^23^. So, additional data on this topic is warranted to illuminate the genetic associations among psychiatric disorders and EF.

**Fig. 1:**
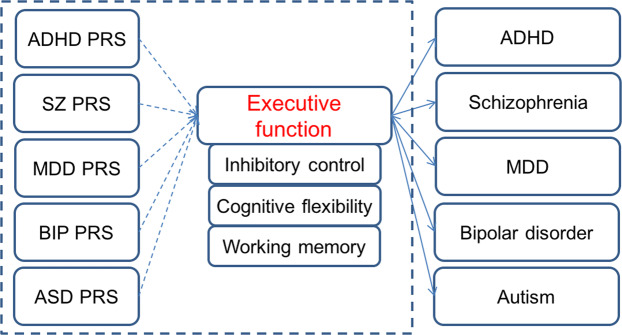
The hypothesis of this study. The solid lines denote the correlation between psychiatric and executive functions at phenotype level. The dotted lines denote the hypothesis to be tested: whether the genetic risk for different psychiatric disorders are associated with different executive dysfunctions. ADHD attention deficit hyperactivity disorder, SZ schizophrenia, MDD major depressive disorder, BIP bipolar disorder, ASD autism, PRS polygenic risk score.

Based on the correlation between psychiatric disorder and executive function at phenotype level, our hypothesis (Fig. 1) is that EF is affected by the genetic risks for multiple psychiatric disorders and it is possible that the genetic risks for different disorders are associated with deficits in specific EF components. In the present study, we examined the genetic associations of five psychiatric disorders (ADHD, MDD, SZ, BIP, ASD) with three executive function components (inhibition, working memory, cognitive flexibility) using polygenic risk scores in a clinically-recruited sample of ADHD children sample. We sought to test our hypothesis and to explore the specific associations between these psychiatric disorders and each EF component. Furthermore, since inhibitory control is a well-replicated deficit of ADHD, we hypothesized that the PRS for inhibitory control would also affect ADHD symptoms and would mediate the effects of disorder-specific PRSs on ADHD symptoms.

## Methods

### Participants

The participants who underwent EF tests included 1147 ADHD patients (954 boys, 83.2%) aged between 6 and 17 years [average (10.1 ± 2.6) years] who were recruited from the Child and Adolescent Psychiatric Outpatient Department of the Peking University Sixth Hospital. All participants met the DSM-IV diagnostic criteria for ADHD. The clinical diagnosis was first made by a senior child and adolescent psychiatrist based on the parent- and teacher-completed ADHD Rating Scale-IV and was then confirmed by a semi-structured interview with the parents and child, using the Chinese version of the Clinical Diagnostic Interview Scale^32,33^. Individuals with major neurological disorders (e.g., epilepsy), SZ, pervasive developmental disorder or intellectual disability (IQ < 70) were excluded. Individuals with comorbidities were not necessarily excluded. In this sample, 349 (29.7%) children had oppositional defiant disorder (ODD), 28 (2.4%) had conduct disorder, 79 (6.7%) had a specific phobia, 18 (1.5%) had a social phobia, 19 (1.6%) had generalized anxiety disorder, 13 (1.1%) had MDD, 6 (0.5%) had BIP, and 178 (15.1%) had a tic disorder. IQ was assessed using the third edition of the Chinese version of the Wechsler Intelligence Scale for Children. Most subjects were drug naive. Only 31 (2.6%) patients had used methylphenidate, and 150 (12.8%) had used Chinese herbal medicine. For individuals who had been medicated, the drugs were washed out for at least 1 month before the patient was tested. All these samples were independent from the samples in our previous ADHD GWAS paper^34^, neither overlapping individuals nor related siblings or cousins. The study was approved by the Institutional Review Board of the Peking University Sixth Hospital. Written informed consent was obtained from the parents of the ADHD probands.

### Executive function tests

From the 1,147 subjects with EF measures, the numbers of ADHD patients for each EF test are shown in Table 1. The details of these tests are as follows.

**Table 1:** Skewness and kurtosis of executive function measures and ADHD symptoms.

	#subjects	Mean (SD)	Skewness (SD)	Kurtosis
Inhibitory control	963	28.72 (17.32)	0.804 (0.079)	3.683 (0.157)
Cognitive flexibility	913	129.98 (97.65)	1.564 (0.081)	3.452 (0.162)
Working memory	1145	4.32 (1.68)	0.632 (0.071)	0.698 (0.143)
ADHD symptoms	1134	11.79 (2.97)	0.176 (0.073)	-0.621 (0.149)

#### Stroop test for inhibitory control

The child and adolescent psychiatrist monitored all the tests conducted on patients. The task included four sessions as described in^35^. At the beginning, thirty stimuli were presented in a 10 × 3 matrix for three cards each sized 21 × 29.7 cm^2^. In the first session, the research participants were asked to read the names of colors (red, green, yellow, and blue) printed in black ink. In the second session, they were asked to name the colored squares (red, green, yellow, and blue). In the third session, the participants were asked to read the color words printed in different colors. In the fourth session, they were asked to name the colors of the ink. The time required to complete each session was recorded. In this study, we used the word interference time, which equals the time required to complete session 4 minus that for session 2, to assess interference inhibition. Higher score indicates worse performance.

#### Trail-Making Test for cognitive flexibility

The test consisted of two sections (A and B), as described in^36^. In section A, numbers from 1 to 25 were randomly scattered on the page, and the participant was asked to connect these numbers sequentially as quickly as possible. In section B, the participant was asked to connect numbers and letters alternately (i.e., 1->A->2->B->3->C, … L->13). When the participant made an error, the investigator pointed out the error immediately before continuing the test. The time to complete section A, T_A_, indicates motor speed and visuo-perceptual abilities, while the time to complete section B, T_B_, is sensitive to working memory and cognitive flexibility. The time difference T_B_−T_A_, which was highly related to T_B_ (*r* = 0.94), minimizes visuo-perceptual and working memory demands, providing a relatively pure indicator of executive control. Therefore, the shifting time = T_B_−T_A_ was the main measure for assessing cognitive flexibility^37^. Higher score indicates worse cognitive flexibility.

#### Digit span test for working memory

The digit span test is a component of the Chinese version of Wechsler Intelligence Scales for Children (C-WISC). In this study, we used the digits backward digit span test to assess the patient’s working memory, as it measures the child’s ability to manipulate verbal information while it is in temporary storage^38^. In this test, the child listens to a sequence of numbers and repeats them in reverse order. The length of each sequence of numbers increases as the child responds correctly. Higher score indicates better working memory.

The phenotype distribution plot showed working memory was normally distributed, but inhibitory control and cognitive flexibility had some outliers. Considering excessive scores on cognitive measures may indicate the participant did not understand the task or something else happened during testing, using the method described in ref. ^39^, we used a loose threshold to remove the samples with inhibitory control or cognitive flexibility below F_L_−4 × (F_U_−F_L_) or above F_U_ + 4 × (F_U_−F_L_), in which F_L_ and F_U_ were the lower and upper fourths of the phenotype. We removed 1 and 2 outliers for inhibitory control and cognitive flexibility respectively. From the 1134 subjects, we also collected ADHD symptoms according to the Clinical Diagnostic Interview Scale^40^. The phenotype distribution plot and related skewness and kurtosis after removing outliers is shown in Fig. S1 and Table 1. We compared the phenotype data with a small group of controls and the result showed ADHD patients had significantly worse performance in shifting time, inhibitory control and working memory than normal controls (Table S1).

For subjects with all three EF measures, we did exploratory factor analysis (EFA) using SPSS 26. EFA method is to identify the underlying relationships between measured variables and get a latent variable to denote common factor of the measured variables. The KMO and Bartlett’s test was significant (*P* < 0.001), which denoted the three EF measures had high correlation and were suitable for EFA. The scree plot showed the first principal component (EF-PC) had eigenvalue >1. Detailed parameters for the EFA model was shown in Fig. S2. The total variance explained by the first component is 56.523%. We used EF-PC to denote the main component among these three EF measures. To test the correlations among the EF measures (including EF-PC) and correlations with ADHD symptoms, two-tailed Pearson correlation was conducted by using SPSS 26.

### Genotyping and imputation

The ADHD patients who underwent executive function tests were genotyped using the InfiniumPsychArray-24 array by CapitalBio Ltd. (Beijing). First, we removed individuals with per-individual autosomal heterozygosity >5 S.D. larger than the mean, individuals without age or sex information, individuals with a per-individual call rate <95% and individuals with the lower call rate in a pair of individuals with proportion identity by descent (IBD) PI_HAT > 0.185. Next, we removed the SNPs with a per-SNP call rate <95%, a Hardy–Weinberg equilibrium test result with *P* < 0.001, or a minor allele frequency<1%. After quality control was performed, 1147 samples with 328,390 SNPs remained.

Principal component analyses (PCA) was conducted using the SNPs with low linkage disequilibrium (LD, MAF > 0.35 and *r*^2^ < 0.05 for each pair of SNPs) that were outside the 5 long-range LD regions^41^ using the EIGENSOFT 4.2 software^42^. The PCA plot for the first two principal components (PCs) was shown in Fig. S3. The top principal component (PC) was significant by the Tracy-Widom test^43^. Genotype imputation was performed using the pre-phasing/imputation stepwise approach implemented in IMPUTE2^44^ and SHAPEIT^45^. The imputation reference set consisted of 2186 phased haplotypes from the full 1000 Genomes Project Integrated Phase 3 Release^46^. Imputed SNPs with a squared correlation between the imputed and true genotypes (rsq) <0.9 or SNPs with minor allele frequency <0.01 were removed. After imputation, 6,064,858 SNPs were used.

### Use of GWAS summary data to derive polygenic risk scores

Summary statistics from GWAS of ADHD^47^, MDD^48^, BIP^49^, SZ, and autism spectrum disorders^50^ were downloaded from the PGC website. These summary data were used as the discovery set to generate the polygenic risk scores for the EF test results. The sample size and number of SNPs for these data sets are in Table S2. Additional details on the demographics for the GWAS summary data are provided in the Supplementary Information.

### Polygenic risk score analysis

The polygenic risk score (PRS) was introduced to summarize the effect of a set of SNPs in a test data set based on the GWAS summary statistics of a discovery dataset. PRSice-2 was used to calculate the results^51^. Before generating the scores, clumping was used to obtain SNPs in linkage equilibrium with an *r*^*2*^ < 0.1 within a 250 bp window. PRSs from the GWAS summary data for five psychiatric disorders were created for each cognitive phenotype using the SNPs selected according to the significance of their association. The *P*-value threshold for significance was set from 0 to 0.5, increasing by 0.00005. The associations between the polygenic profile and the target phenotypes were examined in linear regression models with months (measure of age), sex, IQ and the first two PCs from PCA as covariates. The *P*-value threshold with the largest Nagelkerke’s *r*^*2*^ (variance explained by the PRS) was considered the best-fit threshold. The *P*-value for the linear regression was adjusted by using 10,000 label-swapping (randomly shuffle the phenotype) permutations. Adjusted *P* < 0.05 was considered as significant. To compare the effect of the PRS from different disorders on EF, we did normalization for the PRS under the best *P*-value threshold from PRSice-2 and further ran regression model using the same covariates in R to get the standardized beta coefficient.

### Mediation analysis among PRS, inhibitory control, and ADHD symptoms

Since the inhibitory control is the core deficit of ADHD, the hypothesis is the PRS associated with inhibitory control may could affect the ADHD symptoms too. So, two mediation models were analyzed: (1) ADHD PRS associated with inhibitory control as independent variable (X), inhibitory control as mediator (M), ADHD symptoms as outcome (Y); (2) MDD PRS associated with inhibitory control as independent variable (X), inhibitory control as mediator (M), ADHD symptoms as outcome (Y). The standardized PRS was used for this analysis. The analysis was performed using the model 4 in PROCESS^52^. Both the mediator model and the outcome model included months (measure of age), IQ, sex and two PCs as covariates. Nonparametric bootstrapping with *n* = 10,000 was used to estimate the sampling distribution of the indirect effect. If the confidence interval (CI) of the effect didn’t cross zero, the indirect effect was significant. R package “mediation” was further used to for the mediation analysis to double check the result.

## Results

### Phenotype description for the EF measures and ADHD symptoms

Table 1 shows descriptive statistics for the EF measures and ADHD symptoms. Among the 1,147 individuals, 963, 913, 1145, and 1134 subjects had inhibitory control, cognitive flexibility, working memory and ADHD symptom data, respectively. The distributions of the phenotypes are shown in Fig. S1. One principal component (EF-PC) was extracted from the 911 individuals having all three EF measures. The EF-PC explained 56.5% of the total variance in the exploratory factor model (see Figure S2 for details). All EF measures were significantly correlated with each other (Table 2). Inhibitory control was positively correlated with cognitive flexibility (*r* = 0.337); working memory was negatively correlated with inhibitory control (*r* = −0.267) and cognitive flexibility (*r* = −0.390). As expected, the correlations of EF-PC with the EF measures were higher (*r* = 0.718, 0.776, and −0.759 for inhibitory control, cognitive flexibility, and working memory respectively). All four EF measures were significantly correlated with ADHD symptoms (*r* = 0.153, 0.106, and −0.184 for inhibitory control, cognitive flexibility, and working memory respectively) and IQ (*r* = −0.120, −0.301, and 0.298 for inhibitory control, cognitive flexibility, and working memory respectively), which denoted the EF measures could significantly affect ADHD symptom and IQ; the correlation between ADHD symptoms and IQ was not significant (*r* = 0.037) (Table 2).

**Table 2:** Pearson correlations among phenotypes.

	Inhibitory control	Cognitive flexibility	Working memory	EF-PC	ADHD symptom	IQ
Inhibitory control	–					
Cognitive flexibility	0.337**	–				
Working memory	−0.267**	−0.390**	–			
EF-PC	0.718**	0.776**	−0.759**	–		
ADHD symptoms	0.153**	0.106**	−0.184**	0.204**	–	
IQ	−0.120**	−0.301**	0.298**	−0.327**	−0.037	–

The number of subjects for the correlation analysis among the three EF measures and EF-PC was 911.The number of subjects for the correlation analysis between EF measures and ADHD symptoms was 900. IQ was available for all participants. Asterisks (**) denotes *P*-value <0.01 (two-tailed). EF-PC: principal component for the three EF measures.

### Association of polygenic risk for psychiatric disorders with executive functions

As shown in Table S3, EF-PC was significantly associated with the PRS for MDD (adjusted *P* = 0.023, *r*^*2*^ = 0.581%, coefficient = 0.0760 (se = 0.023)), ADHD (adjusted *P* = 0.036, *r*^*2*^ = 0.486%, coefficient = 0.0704 (se = 0.023)), and BIP (adjusted *P* = 7.70E−03, *r*^*2*^ = 0.642%, coefficient = 0.080 (se = 0.023)).

**Fig. 2:**
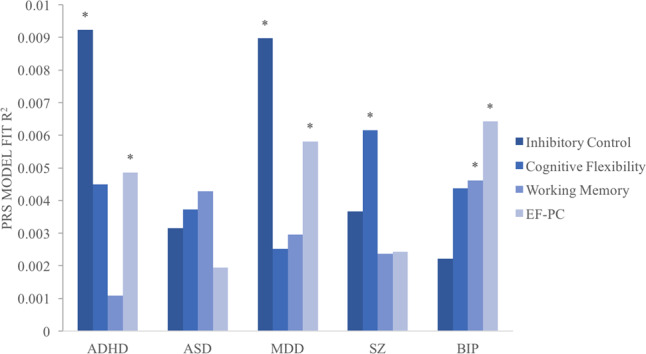
The proportion of variance in the three executive function measures and their principal component (EF-PC) explained by the PRSs of psychiatric disorders. Asterisk (*) denotes the adjusted *P*-value <0.05. ADHD attention deficit hyperactivity disorder, SZ schizophrenia, MDD major depressive disorder, BIP bipolar disorder, ASD autism, EF-PC principal component for the three EF measures.

We further explored the association of each EF component with the genetic risk for psychiatric disorders (Fig. 2, Table S4). The PRS for ADHD was significantly associated with inhibitory control (adjusted *P* = 0.018, *r*^*2*^ = 0.923%, coefficient = 1.6922 (se = 0.5225)); the PRS for SZ was significantly associated with cognitive flexibility (adjusted *P* = 0.034, *r*^*2*^ = 0.615%, coefficient = 7.7142 (se = 2.6781)); the PRS for MDD was significantly associated with inhibitory control (adjusted *P* = 0.0313, *r*^*2*^ = 0.897%, coefficient = 1.6559 (se = 0.5188)); the PRS for BIP was significantly associated with working memory (adjusted *P* = 0.0416, *r*^*2*^ = 0.462%, coefficient = −0.1152 (se = 0.0391)); and the association of PRS for ASD with all three EF components did not pass the permutation correction.

Since both the ADHD and MDD PRSs were associated with inhibitory control, we constructed a multivariable regression model with standardized ADHD PRS, MDD PRS, their interaction and other covariates. The model showed *P* = 2.41E−03 for ADHD PRS and *P* = 2.86E−03 for MDD PRS, and the interaction was not significant (Table S5). Thus, each of these PRS were uniquely associated with inhibitory control.

### Correlation and mediation analysis with ADHD symptoms

**Fig. 3:**
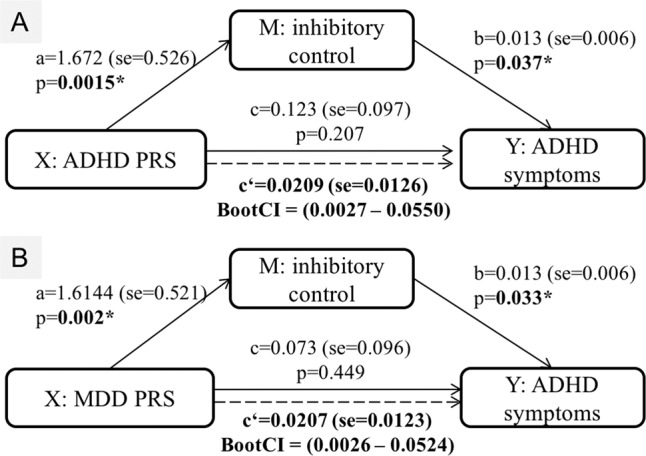
Mediation analysis results for PRS, inhibitory control and ADHD symptoms. (**a**) ADHD PRS as independent variable (X), inhibitory control as mediator (M), ADHD symptoms as outcome (Y); (**b**) MDD PRS as independent variable (X), inhibitory control as mediator (M), ADHD symptoms as outcome (Y). Path a is X variable predict M, path b is M variable predict Y, path c is the direct effect of X on Y, path c’ is the indirect effect of X on Y through the mediation of M. BootCI is the confidence interval (CI) of the indirect effect from bootstrap.

The ADHD PRS had a trend to be associated with ADHD symptoms but did not pass the multiple corrections (*P*-value = 0.0194, adjust *P* = 0.1828, *r*^*2*^ = 0.46%, beta = 0.2028 (se = 0.0867)). The ASD PRS was significantly associated with ADHD symptoms (*P*-value = 2.549E−03, adjust *P* = 0.038, *r*^*2*^ = 0.765%, beta = 0.2618 (se = 0.0866)). The PRSs of other disorders were not associated with ADHD symptoms (Table S6). To assess the indirect effect of the PRSs associated with inhibitory control on ADHD symptoms, we conducted mediation analyses. The ADHD PRS associated with inhibitory control had no direct effect on ADHD symptoms, but the indirect effect through the mediation of inhibitory control was significant (effect = 0.0209 (se = 0.0126), BootCI = (0.0027–0.0550)) (Fig. 3A). The MDD PRS associated with inhibitory control also showed an indirect effect on ADHD symptoms through the mediation of inhibitory control (effect = 0.0207 (se = 0.0123), BootCI = (0.0026–0.0524)) (Fig. 3b). The result from the “mediation” R package also obtained the same results (Table S7).

## Discussion

This study used polygenic risk score analyses to explore the shared and specific genetic associations between psychiatric disorders and executive functions. The PRSs for MDD, ADHD, and BIP were associated with our overall measure of EF. These results agree with the idea that executive dysfunction is a transdiagnostic phenotype^53^. On the other hand, we also found that some EF components associated with PRSs for different disorders: the ADHD and MDD PRSs were associated with inhibitory control; the PRS for BIP was associated with working memory, and that for SZ was associated with cognitive flexibility.

Dysfunction of inhibitory control and working memory in individuals with ADHD has been reported by many studies^40,54^. Some studies found that the PRS for ADHD was associated with working memory^25–27^, but in this study, we did not identify the association of ADHD PRS with working memory, which may indicate the genetic basis of working memory was more complex and the current sample size was not large enough to catch the association of ADHD PRS with working memory. The previous papers did not find the association of ADHD PRS with inhibitory control in community recruited populations^25–27^, but we found the ADHD PRS was associated with inhibitory control as measured by the variance of word interference time. The divergent results might be due to the different target sample because, compared with population samples, our ADHD sample would be expected to be enriched for the disinhibition. Mediation analyses indicated that the ADHD PRS contributed to ADHD symptoms through the mediation effect of inhibitory control. This result suggests that ADHD risk variants might affect inhibitory control, which leads to ADHD symptoms. This result is consistent with theoretical models that posit inhibitory control as a core deficit of ADHD patients.

Depression and ADHD often co-occur in clinical populations. ADHD symptoms are very common among MDD patients^55^, while ADHD patients have higher rates of MDD^56^. Inhibitory dysfunction has been reported, not only for patients with ADHD, but also for patients with MDD, as measured by the stop signal and Stroop inhibition tasks^57^. The comorbidity between ADHD and MDD can partially be explained by their shared cognitive impairments and shared genetic variants. The genetic correlation between ADHD and MDD has been reported by the Brainstorm Consortium and the ADHD-PGC results^17,47^. The present study found that the MDD PRS was also associated with inhibitory control. Although prior studies show the genetic risk for MDD to be associated with general cognitive ability^21^, ours is the first study that reports an association with inhibitory control. Both results are in the same direction, with higher genetic risk associated with worse performance. Furthermore, the mediation analysis showed that the MDD PRS also had an indirect on ADHD symptoms through the mediation of inhibitory control. These results again suggested a shared genetic risk between these two disorders that may be mediated by the inhibitory control.

Although some study identified the association of inhibitory control with SZ and BIP^58^, we didn’t find their association but the association of BIP PRS with working memory and SZ PRS with cognitive flexibility. Working memory impairment is a characteristic that BIP shares with ADHD. Brown et al. reported hypoactivity in the frontal and parietal regions in individuals with ADHD and those with BIP compared with controls when they performed a working memory task^59^. *CACNA1C*, a common risk gene for five major psychiatric disorders, has been associated with spatial working memory in BIP^60^. In this study, the PRS for BIP was associated with decreased working memory, providing further evidence for the genetic correlation between BIP, working memory and ADHD. Impairment of cognitive flexibility has been found in SZ^61^. In our study, the PRS for SZ was associated with cognitive flexibility in ADHD children. Although Vitiello et al. did not find that ADHD increased the risk for psychotic symptoms^62^, a significant genetic correlation between ADHD and SZ was reported^17,47^. Our result suggests that impaired cognitive flexibility has a shared genetic basis in ADHD and SZ, which is in the same direction as the association with general cognitive function.

The relationship of cognition with ASD is complex. Genetic correlation analysis^17,50^ and PRS analysis in the general population^23^ showed that the polygenic risk for ASD was associated with *higher* general cognition or intelligence. In contrast, the PRS analysis for EF showed the opposite effect such that high PRS for general intelligence was associated with worse social problems^28^. Grove et al. identified a positive genetic correlation between ADHD and ASD^50^ and both of ADHD and ASD were reported to have executive impairments. In this study, the ASD PRS was significantly associated with ADHD symptoms, but not EF. One possible explanation is that EF is not a core impairment in ASD, for which social communication and repetitive behavior were core symptoms that might be directly correlated with the genetic risk of the disorder. ASD may express different features of EF deficits from ADHD^63^. Another plausible explanation is the relatively low number of genome-wide significant signals in the GWAS of autism^50^.

Compared with previous PRS analyses for cognition and psychiatric disorders, a main difference with this study is the age of our sample and how it was ascertained. Most prior studies used data from community-based populations; we used data from ADHD patients. The distribution of EF may differ in these populations. The samples used in this study likely have ADHD-specific features (e.g., worse executive functions) that differ from the general population, which might facilitate the finding of shared cognition deficit with other psychiatric disorders by using PRS analysis. Another difference was the age of the samples. Most prior studies used adolescent or adult samples, whose cognitive development differs with children’s. The association of genetic risk of some disease with children’s cognition may not be consistent for adults. For example, ADHD related impairment that exist in childhood can disappear or attenuate in late adolescence or adults^64^.

This study has some limitations. The sample sizes for the EF measures were small. Replication in larger samples is needed. Second, the GWAS summary data for the PRS calculations in this study were from European individuals. Cross-ethnic PRS analyses are affected by the different LD structures and allele frequencies in different ethnicities^65^, which reduces the power to find associations. We also attempted to analyze the association of PRSs for the Chinese ADHD GWAS data^34^ with EF. Since the sample size of the Chinese ADHD GWAS is small, there was no significant result for all EF components (data not shown). A larger sample size is important for the PRS definition to address a comprehensive genetic component. As estimated by Martin et. al.^66^, applying GWAS summary statistics from the European population to other ancestries will reduce prediction accuracy. If using Chinese population GWAS data with the same sample size, the effect size might be bigger.

In conclusion, by using polygenic risk score analyses, we found that the polygenic risk for different psychiatric disorders (ADHD, BIP, MDD, SZ) showed distinct effects on executive function domains. Although all psychiatric disorders had general cognition dysfunction, the specific impairments of the EF components may be different. The results of this study helped to identify the core cognitive impairment that most associated with the genetic risk for each mental disorder, which may further help elucidate the risk genes and motivate efforts to develop EF measures as a diagnostic marker and future treatment target.

## Supplementary information

Supplementary Information
