# Supplementary material for: Shared polygenic risk for ADHD, executive dysfunction and other psychiatric disorders
Source: Transl Psychiatry. 2020 Jun 9;10:182. doi: 10.1038/s41398-020-00872-9 (PMC7283259; doi:10.1038/s41398-020-00872-9)
Supplement: Supplementary file 1 — Supplementary Information [file 41398_2020_872_MOESM1_ESM.docx]

**Supplementary Materials**

**Shared Polygenic Risk for ADHD, Executive Dysfunction and Other Psychiatric Disorders**

Suhua Chang^1,2^, Li Yang PhD^1,*^, Yufeng Wang^1^, Stephen V. Faraone ^3,*^

*^1^* *Peking University Sixth Hospital, Peking University Institute of Mental Health, NHC Key Laboratory of Mental Health (Peking University), National Clinical Research Center for Mental Disorders (Peking University Sixth Hospital).*

*^2^Research Unit of Diagnosis and Treatment of Mood Cognitive Disorder (2018RU006), Chinese Academy of Medical Sciences.*

*^3^Departments of Psychiatry and of Neuroscience and Physiology, SUNY Upstate Medical University, Syracuse NY, USA.*

Corresponding authors:

Stephen V. Faraone, Departments of Psychiatry and of Neuroscience and Physiology, SUNY Upstate Medical University, Syracuse NY, USA. Tel: 315 464-3113, Fax: 315-849-1839, E-mail: sfaraone@childpsychresearch.org;

Li Yang, Institute of Mental Health, Peking University, Beijing 100191, China. Tel: 86-10-62350880, Fax: 86-10-62350880, Email: [yangli_pkuimh@bjmu.edu.cn](mailto:yangli_pkuimh@bjmu.edu.cn).

**Running title**: Association of PRS of psychiatric disorders with EF

**Supplementary Methods**

**GWAS summary data as discovery data to derive polygenic risk scores**

Summary statistics from PGC ADHD [^1^](#_ENREF_1), major depressive disorder (MDD) [^2^](#_ENREF_2), bipolar disorder (BIP) [^3^](#_ENREF_3), schizophrenia (SZ) [^4^](#_ENREF_4), and autism spectrum disorders (ASD) [^5^](#_ENREF_5) were downloaded from the PGC website (https://www.med.unc.edu/pgc/results-and-downloads). The sample ancestry description is as below and in Table S1:

**ADHD**: ADHD GWAS summary data is from the latest PGC ADHD GWAS paper [^1^](#_ENREF_1). The summary data for the worldwide GWAS was used, including all samples (20,183 cases and 35,191 controls).

**MDD**: MDD GWAS summary data is from the largest MDD meta-analysis (135,458 cases and 344,901 controls), which included seven cohorts. The samples come from various countries, mainly are European ancestry.

**BIP**: The summary data used BDvsCONT.sumstats.gz file. It is calculated from a BIP cases (n=20,129) against an independent bipolar specific set of controls (n=21,524). All samples are European ancestry.

**SZ**: SZ GWAS summary data is from the PGC paper with 108 significant loci. The sample size is 36,989 cases and 113,075 controls. The samples are 46 of European and three of east Asian ancestry.

**ASD**: This is the GWAS results file from the meta-analysis of ASD by the Lundbeck Foundation Initiative for Integrative Psychiatric Research (iPSYCH) and the Psychiatric Genomics Consortium (PGC) released in November 2017. iPSYCH-PGC_ASD_Nov2017.gz: Full ASD GWAS meta-analysis of samples of European ancestry (18,382 cases, 27,969 controls).

**Polygenic risk score analysis**

The polygenic risk score (PRS) was introduced to summarize the effect of a set of SNPs in a test data set based on the GWAS summary statistics of a discovery dataset. PRSice-2 was used to calculate the results[^6^](#_ENREF_6). Before generating the scores, clumping was used to obtain SNPs in linkage equilibrium with an *r^2^*<0.1 within a 250 bp window. PRSs from the GWAS summary data for five psychiatric disorders were created for each cognitive phenotype using the SNPs selected according to the significance of their association. The *P*-value threshold for significance was set from 0 to 0.5, increasing by 0.00005. The associations between the polygenic profile and the target phenotypes were examined in linear regression models with age, sex, IQ and the first two PCs as covariates. The *P*-value threshold with the largest Nagelkerke’s *r^2^* (variance explained by the PRS) was considered the best-fit threshold and the p-value of association under the best p-value threshold was *P_0_*. The *P*-value for the linear regression was adjusted by using 10,000 label-swapping permutations, which randomly shuffled the phenotype (the EF measure) and repeat the procedure as above to obtain the p-value of association under the null (*P_null_*), repeat the permutation *N* = 10000 times to obtain the adjusted *P* = (1+times of *P_null_*<*P_0_*)/(*N*+1). Adjusted *P*<0.05 was considered as significant. The permutation based p-value could control the Type 1 error, but the observe phenotypic variance explained *r^2^* may be affected by overfitting. By now, for most of PRS studies, the *r^2^* is still too low to have real meaning. We mainly focus on the significance of the association. To compare the effect of the PRS from different disorders on EF, we did normalization for the PRS under the best P-value threshold from PRSice-2 and further ran regression model using the same covariates in R to get the standardized beta coefficient.

**Table S1** Executive functioning differences between ADHD patients and normal controls.

|  | **Source** | **Mean** | **SE** | **N** | **F** | ***P*-value** |
| --- | --- | --- | --- | --- | --- | --- |
| Working memory | Case | 4.32 | 1.68 | 1145 | 85.6770 | 8.726E-20 |
|  | Control | 5.45 | 1.63 | 129 |  |  |
| Inhibitory control | Case | 28.72 | 17.32 | 963 | 25.2685 | 5.802E-07 |
|  | Control | 21.26 | 9.27 | 164 |  |  |
| Cognitive flexibility | Case | 129.98 | 97.65 | 913 | 10.4210 | 1.284E-03 |
|  | Control | 92.50 | 71.54 | 161 |  |  |

The controls were recruited from local elementary schools. Among the 187 controls, 112 (59.9%) were boys, average age was 9.74 (SD=1.74). Their parents completed the ADHD Rating Scale-IV (ADHD RS-IV) to exclude ADHD. Major psychiatric disorders, family history of psychosis, severe physical diseases, and substance abuse were also excluded according to a medical history report form. All the executive function tests were the same with ADHD patients. The differences in executive function measures between ADHD patients and controls were analyzed using the analysis of covariance (ANCOVA), with IQ, sex, and age as covariates.

**Table** **S2** Data sets used for polygenic risk score calculations for five psychiatric disorders.

| **Data set** | **Reference** | **Sample size** | **Number of SNPs** |
| --- | --- | --- | --- |
| ADHD | [^1^](#_ENREF_1) | 20,183 cases and 35,191 controls | 6,517,325 |
| MDD | [^2^](#_ENREF_2) | 135,458 cases and 344,901 controls | 13,554,550 |
| BIP | [^3^](#_ENREF_3) | 20,129 cases and 21,524 controls | 8,958,990 |
| SZ | [^4^](#_ENREF_4) | 36,989 cases and 113,075 controls | 9,444,230 |
| ASD | [^5^](#_ENREF_5) | 18,382 cases and 27,969 controls | 6,517,325 |

ADHD: attention deficit hyperactivity disorder, MDD: major depressive disorder, BIP: bipolar disorder, SZ: schizophrenia, ASD: autism.

**Table S3** The association of PRSs for psychiatric disorders with principal component for the three executive function measures (EF-PC).

| **Discovery** | **Target** | **Threshold** | **PRS.R2** | **Beta** | **SE** | ***P*** | **Num SNP** | **Adjusted *P*** |
| --- | --- | --- | --- | --- | --- | --- | --- | --- |
| ADHD | EF-PC | 0.00075 | 0.00486 | 0.07037 | 0.02333 | 2.628E-03 | 1277 | **0.0362** |
| ASD | EF-PC | 0.00105 | 0.00194 | 0.04469 | 0.02355 | 5.805E-02 | 1131 | 0.4432 |
| MDD | EF-PC | 0.00725 | 0.00581 | 0.07600 | 0.02303 | 1.003E-03 | 5545 | **0.0228** |
| SZ | EF-PC | 0.1073 | 0.00243 | 0.04975 | 0.02338 | 3.362E-02 | 33673 | 0.1952 |
| BIP | EF-PC | 0.0346 | 0.00642 | 0.08022 | 0.02310 | 5.382E-04 | 14306 | **7.699E-03** |

ADHD: attention deficit hyperactivity disorder; ASD: autism; MDD: major depressive disorder; SZ: schizophrenia; BIP: bipolar disorder; EF-PC: principle component of executive function. Beta is the coefficient. SE is the standard error. The coefficient and standard error are standardized.

**Table S4** The association of PRS for psychiatric disorders with three executive function measures.

| **Discovery** | **Target** | **Threshold** | **PRS.R^2^** | **Beta** | **SE** | ***P*** | **Num SNP** | | **Adjusted *P*** | |
| --- | --- | --- | --- | --- | --- | --- | --- | --- | --- | --- |
| ADHD | Inhibitory control | 0.0016 | 0.00923 | 1.69216 | 0.52248 | 1.242E-03 | 2060 | **0.0183** | |  |
| ADHD | Cognitive flexibility | 0.00055 | 0.00449 | 6.63016 | 2.69491 | 0.0141 | 1053 | 0.1397 | |  |
| ADHD | Working memory | 0.4531 | 0.00108 | 0.05591 | 0.03930 | 0.1551 | 64000 | 0.7354 | |  |
| ASD | Inhibitory control | 0.0001 | 0.00315 | -0.97971 | 0.51972 | 0.0597 | 232 | | 0.4406 | |
| ASD | Cognitive flexibility | 0.00105 | 0.00373 | 6.05945 | 2.70551 | 0.0254 | 1131 | | 0.2393 | |
| ASD | Working memory | 0.299 | 0.00428 | 0.11127 | 0.03924 | 4.654E-03 | 50741 | | 0.0616 | |
| MDD | Inhibitory control | 0.0008 | 0.00897 | 1.65587 | 0.51882 | 1.461E-03 | 1255 | | **0.0313** | |
| MDD | Cognitive flexibility | 0.1154 | 0.00251 | 4.82758 | 2.62812 | 0.0666 | 32952 | | 0.5666 | |
| MDD | Working memory | 0.0055 | 0.00296 | -0.09216 | 0.03913 | 0.0187 | 4620 | | 0.2474 | |
| SZ | Inhibitory control | 0.1779 | 0.00367 | 1.05575 | 0.51849 | 0.0420 | 42440 | | 0.2316 | |
| SZ | Cognitive flexibility | 0.1742 | 0.00615 | 7.71419 | 2.67805 | 4.063E-03 | 42042 | | **0.0335** | |
| SZ | Working memory | 0.00025 | 0.00236 | 0.08233 | 0.03920 | 0.0359 | 2060 | | 0.2067 | |
| BIP | Inhibitory control | 0.0015 | 0.00221 | -0.81774 | 0.51814 | 0.1148 | 1961 | | 0.6181 | |
| BIP | Cognitive flexibility | 0.0378 | 0.00438 | 6.44920 | 2.65470 | 0.0153 | 15192 | | 0.1496 | |
| BIP | Working memory | 0.0513 | 0.00462 | -0.11518 | 0.03908 | 3.270E-03 | 18372 | | **0.0416** | |

ADHD: attention deficit hyperactivity disorder; ASD: autism; MDD: major depressive disorder; SZ: schizophrenia; BIP: bipolar disorder. Beta is the coefficient. SE is the standard error. The coefficient and standard error are standardized.

**Table S5** Multivariable regression model for inhibitory control predicted by ADHD PRS, MDD PRS and their interaction as well as other covariates.

|  | **Estimate** | **Std.Error** | **t value** | **Pr(>\|t\|)** |
| --- | --- | --- | --- | --- |
| Sex | -0.28589 | 1.39202 | -0.205 | 0.83732 |
| months | -0.21065 | 0.01684 | -12.508 | < 2.00E-16*** |
| PC1 | -19.57404 | 17.82049 | -1.098 | 0.27231 |
| PC2 | 6.52316 | 17.17995 | 0.38 | 0.70426 |
| IQ | -0.11784 | 0.03728 | -3.161 | 0.00162** |
| ADHD_PRS | 1.58968 | 0.52258 | 3.042 | 0.00241** |
| MDD_PRS | 1.54906 | 0.51806 | 2.99 | 0.00286** |
| ADHD_PRS*MDD_PRS | -0.06196 | 0.51072 | -0.121 | 0.90346 |

PC1 and PC2 are two principal components from the population stratification analysis, which were used as covariates as well as sex, age (use months to measure the age) and IQ.

**Table S6** The association of PRS for psychiatric disorders with ADHD symptoms.

| **Discovery** | **Target** | **Threshold** | **PRS.R2** | **Beta** | **SE** | ***P*** | **Num SNP** | **Adjusted *P*** |
| --- | --- | --- | --- | --- | --- | --- | --- | --- |
| ADHD | ADHD symptoms | 0.1038 | 0.00460 | 0.20279 | 0.08665 | 0.0194 | 30612 | 0.1828 |
| ASD | ADHD symptoms | 0.0306 | 0.00765 | 0.26180 | 0.08657 | 2.549E-03 | 12547 | **0.0377** |
| MDD | ADHD symptoms | 0.0421 | 0.00194 | -0.13118 | 0.08644 | 0.1294 | 18032 | 0.8058 |
| SZ | ADHD symptoms | 5.00E-05 | 0.00174 | -0.12433 | 0.08653 | 0.1510 | 1180 | 0.5767 |
| BIP | ADHD symptoms | 0.2619 | 0.00160 | 0.11964 | 0.08673 | 0.1680 | 45510 | 0.7715 |

ADHD: attention deficit hyperactivity disorder; ASD: autism; MDD: major depressive disorder; SZ: schizophrenia; BIP: bipolar disorder; EF-PC: principle component of executive function. Beta is the coefficient. SE is the standard error. The coefficient and standard error are standardized.

**Table S7** Mediation analysis result using R package “mediation”.

| **Models** | **Model Parameters** | **Estimate** | **95% CI Lower** | **95% CI Upper** | **p-value** |
| --- | --- | --- | --- | --- | --- |
| X: ADHD PRS M: inhibitory control Y: ADHD symptoms | ACME | 0.0209 | 0.0006 | 0.05 | **0.042*** |
|  | ADE | 0.1230 | -0.0728 | 0.31 | 0.212 |
|  | Total Effect | 0.1440 | -0.0534 | 0.33 | 0.155 |
|  | Prop. Mediated | 0.1453 | -0.8305 | 1.14 | 0.182 |
| X: MDD PRS M: inhibitory control Y: ADHD symptoms | ACME | 0.0207 | 0.0016 | 0.05 | **0.03*** |
|  | ADE | 0.0730 | -0.1118 | 0.25 | 0.45 |
|  | Total Effect | 0.0937 | -0.0904 | 0.28 | 0.33 |
|  | Prop. Mediated | 0.2210 | -1.7297 | 2.26 | 0.34 |

ACME: Average Causal Mediation Effects, ADE: Average Direct Effects.

**Figure S1** Histogram of three executive function measures (inhibitory control, cognitive flexibility, working memory) and ADHD symptoms.


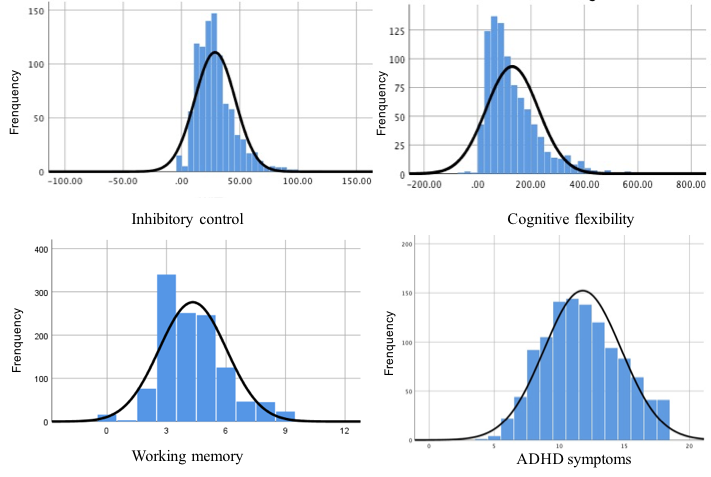


**Figure S2** Exploratory factor analysis results for the three EF measures. KMO and Bartlett’s test was significant, which denoted the three EF measures had high correlation and were suitable for the exploratory factor analysis. One component with eigenvalue > 1 in the scree plot was selected. The total variance explained by the component is 56.5%.

**
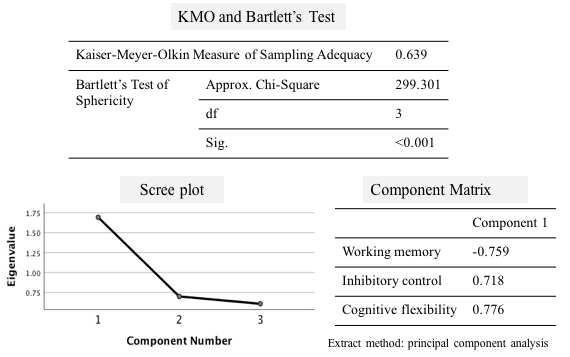
**

**Figure S3** Plot for the first two principal components (PCs) from the principal component analysis (PCA) for the genotype data. The plot showed the samples were homogeneous.


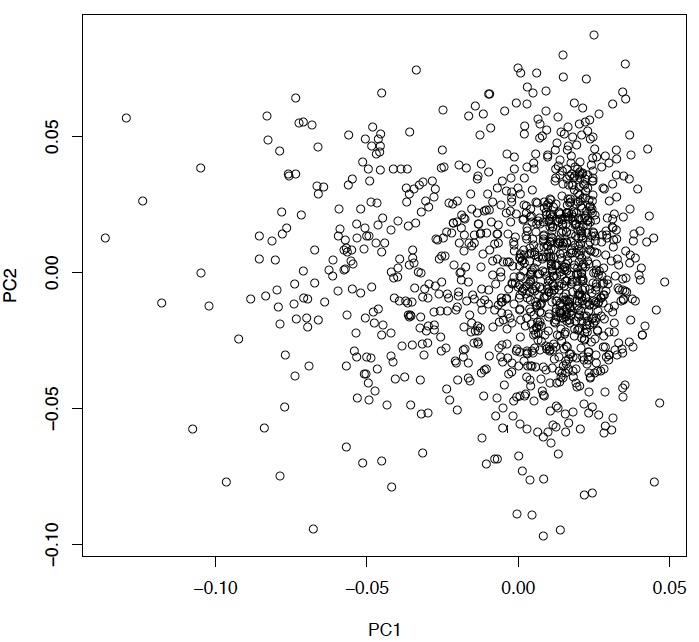


**References**

1. Demontis D. *et al.* Discovery of the first genome-wide significant risk loci for attention deficit/hyperactivity disorder. *Nat Genet* 2019; **51**(1)**:** 63-75.

2. Wray N.R. *et al.* Genome-wide association analyses identify 44 risk variants and refine the genetic architecture of major depression. *Nat Genet* 2018; **50**(5)**:** 668-681.

3. Bipolar D, Schizophrenia Working Group of the Psychiatric Genomics Consortium. Genomic Dissection of Bipolar Disorder and Schizophrenia, Including 28 Subphenotypes. *Cell* 2018; **173**(7)**:** 1705-1715 e1716.

4. Schizophrenia Working Group of the Psychiatric Genomics C. Biological insights from 108 schizophrenia-associated genetic loci. *Nature* 2014; **511**(7510)**:** 421-427.

5. Grove J. *et al.* Identification of common genetic risk variants for autism spectrum disorder. *Nat Genet* 2019; **51**(3)**:** 431-444.

6. Euesden J, Lewis CM, O'Reilly PF. PRSice: Polygenic Risk Score software. *Bioinformatics* 2015; **31**(9)**:** 1466-1468.
